# Effects of Ultrafine Bubble Water on Gut Microbiota Composition and Health Markers in Rats

**DOI:** 10.3390/nano15151193

**Published:** 2025-08-05

**Authors:** John Nicholas Jackowetz, Carly S. Hanson, Minto Michael, Kiriako Tsoukalas, Cassandra Villanueva, Peter A. Kozak

**Affiliations:** Hydrosome Labs LLC, 640 Blackhawk Drive, Westmont, IL 60559, USA; chanson@hydrosomelabs.com (C.S.H.); mmichael@hydrosomelabs.com (M.M.); ktsoukalas@hydrosomelabs.com (K.T.); cvillanueva@hydrosomelabs.com (C.V.); pkozak@hydrosomelabs.com (P.A.K.)

**Keywords:** ultrafine bubbles, nanobubbles, gut microbiota, inflammation, short chain fatty acids, metabolic health

## Abstract

Ultrafine bubbles (UFBs) represent an emerging technology with unique physicochemical properties. This study investigated the effects of air-filled UFBs infused in drinking water on gut microbiota composition and the associated health markers in Sprague Dawley rats over a 12-week period. Using a two-phase design, UFB concentration was increased from 1.7 × 10^6^ to 6.5 × 10^9^ UFBs/mL at week 7 to assess dose-dependent effects. Administration of UFBs in drinking water induced significant shifts in gut microbiome populations, characterized by increased Bacteroidetes (+122% weeks 8–12) and decreased Firmicutes (−43% weeks 8–12) compared to controls. These microbial shifts coincided with enhanced short-chain fatty acid production (butyrate +56.0%, *p* ≤ 0.001; valerate +63.1%, *p* ≤ 0.01) and reduced inflammatory markers (TNF-α −84.0%, *p* ≤ 0.05; IL-1β −41.0%, *p* ≤ 0.05; IL-10 −69.8%, *p* ≤ 0.05). UFB effects demonstrated systematic concentration-dependent threshold responses, with 85.7% of parameters exhibiting directional reversals between low (1.7 × 10^6^ UFBs/mL) and high (6.5 × 10^9^ UFBs/mL) concentration phases rather than linear dose–response relationships. The systematic nature of these threshold effects, with 71.4% of parameters achieving statistical significance (*p* ≤ 0.05), indicates concentration-dependent biological mechanisms rather than random effects on gut biology. Despite current metagenomic techniques identifying only 25% of the total gut microbiome, the observed changes in characterized species and metabolites demonstrate UFB technology’s therapeutic potential for conditions requiring microbiome modulation, providing new insights into UFB influence on complex biological systems.

## 1. Introduction

Ultrafine bubbles (UFBs) are small gas-filled cavities with diameters of less than one micron but generally between 100 and 300 nm [1,2,3,4,5]. The UFBs were formerly known as nanobubbles before receiving an official nomenclature and definition from the International Organization for Standardization (ISO) in 2017 (ISO-20480-1:2017) [6]. UFBs possess unique properties that set them apart from their larger counterparts [i.e., micro- (1–10 µm) and macro-bubbles (>10 µm)]. Notably, UFBs’ high surface-to-volume ratio endows them with enhanced surface area and activity, enabling efficient mass transfer, and improved solubility of gases [7,8,9]. Additionally, UFBs exhibit exceptional stability due to their negative surface charge and reduced buoyancy, which prolong their lifespan in liquids [7,10,11,12]. Moreover, their small size and charge improve transport, making UFBs ideal carriers for delivering active ingredients and nutrients in various applications, including cosmetics, pharmaceuticals, and agriculture [7,13,14,15].

Recent advances in UFB generation technology have led to an increased interest in their biological applications [16,17], particularly in the realm of gut health [18]. This increased attention coincides with increased consumer awareness regarding the gut microbiome’s role in overall health. The gut microbiome, comprising trillions of microorganisms living in our digestive tract, plays a crucial role in everything from immune function and metabolism to mental health and disease prevention [19]. Research has shown that the diversity and balance of these microbial communities can significantly impact overall health outcomes, with imbalances being linked to conditions ranging from inflammatory bowel disease and obesity to anxiety and depression [20]. The gut–brain axis, mediated through microbiome-derived metabolites and immune system interactions, has emerged as a key pathway through which gut bacteria influence systemic health [21]. In particular, Firmicutes and Bacteroidetes represent the two dominant bacterial phyla in a healthy human gut, typically comprising 80–90% of the microbial community [22]. The Firmicutes-to-Bacteroidetes (F/B) ratio serves as a key indicator of gut health, with elevated ratios historically associated with obesity and metabolic dysfunction, though recent evidence suggests this relationship is more complex than initially proposed [23].

Among the crucial metabolites produced by gut bacteria, short-chain fatty acids (SCFAs) have garnered particular attention as they serve as an energy source for gut bacteria [24] and intestinal cells [25] and regulate gut barrier integrity [26]. High SCFA production is associated with positive health outcomes, including increased beneficial bacterial growth and reduced pathogenic bacteria linked to several diseases and health deficiencies [27]. The most abundant SCFAs—acetate, propionate, and butyrate—play distinct roles in maintaining gut health, with butyrate particularly important as the primary energy source for colonocytes [28]. Additionally, inflammatory markers such as cytokines are of interest, as elevated cytokine levels are linked with inflammatory and autoimmune diseases, including irritable bowel disease (IBD), arthritis, cardiovascular disease, and diabetes [29]. The balance between pro-inflammatory and anti-inflammatory cytokines is crucial for maintaining immune homeostasis and gut barrier function [29].

While the importance of maintaining healthy gut microbiota is well established, developing effective interventions remains challenging. Current efforts to boost gut microbiome biodiversity, increase SCFA production, and reduce inflammatory markers face significant hurdles, including consumer acceptance, regulatory and safety concerns, and the need for scientifically validated products [30]. Previous research has demonstrated that dietary interventions can effectively modulate gut microbiota to improve metabolic outcomes, with oat phenolic compounds and β-glucan showing synergistic effects on hyperlipidemia through microbiome-mediated pathways [31,32]. This has sparked interest in novel technologies that are both consumer-friendly and compliant with regulatory standards, while effectively enhancing gut microbiome health markers.

Despite the promising physicochemical properties of UFBs and preliminary evidence suggesting their potential to influence biological systems, a significant knowledge gap exists regarding the specific effects of UFB water on gut microbiota composition and associated health markers, as well as the dose-dependent nature of these effects. Previous research by Guo et al. [33] provided initial insights into UFB water’s potential to alleviate obesity-associated markers through microbiome modulation, but comprehensive investigations of UFBs’ effects on gut microbiota composition, SCFA production, and inflammatory marker expression across different concentrations remains limited.

To address this gap, we conducted a preclinical evaluation of air-filled UFB water administered to Sprague Dawley rats over a 12-week period, with a concentration increase at the midpoint to assess dose-dependent effects. We hypothesized that UFB water would (1) induce beneficial shifts in gut microbiota composition, particularly in the Firmicutes-to-Bacteroidetes ratio; (2) enhance the production of beneficial SCFAs; (3) reduce inflammatory marker expression; and (4) demonstrate concentration-dependent biological responses. This study aims to provide deeper insights into the potential mechanisms by which UFB water influences gut health, thereby establishing a foundation for future therapeutic applications of this emerging technology.

## 2. Materials and Methods

### 2.1. Experimental Design and Animal Housing

**Figure 1:**
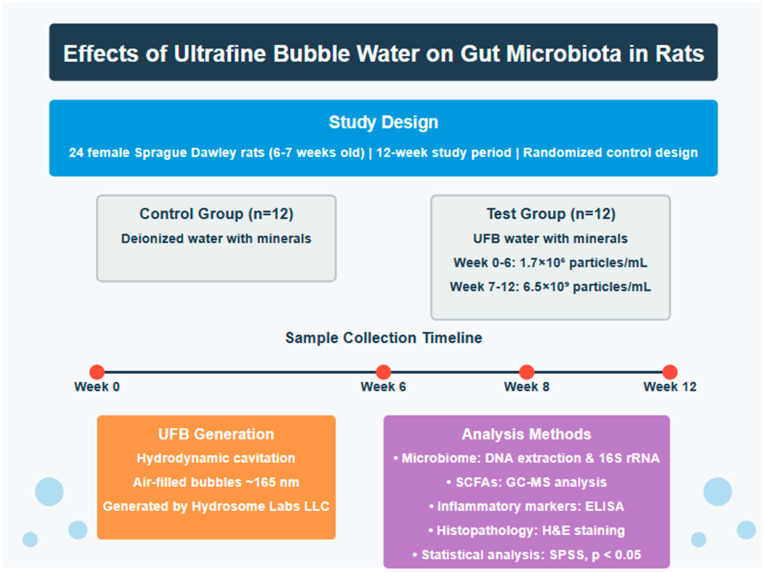
Overview of the study experimental design.

Twenty-four female Sprague Dawley rats (160 ± 20 g, 6–7 weeks old) were housed in individually ventilated cages (IVC; Orchid Scientific & Innovative India Pvt. Ltd., Nashik, India) under controlled conditions (Figure 1). Environmental parameters were maintained at 20 °C ± 3 °C, a relative humidity between 30 and 70%, a 12 h light/dark cycle, and with a minimum of 15 fresh air changes per hour. The animals were housed individually with sterilized polycarbonate cages covered with stainless steel grid top mesh. Autoclaved corn cob was used as bedding material, with cage changes performed at least weekly to avoid ammonia buildup. The animals received a standard laboratory chow diet (Teklad Global 16% Protein Rodent Diet, Envigo) and water ad libitum. The study was approved by the Institutional Animal Ethics Committee (IAEC Protocol No: UH/IAEC/MS/21/03/2024/06) and conducted according to CCSEA guidelines (Registration No: 151/1999/CPCSEA/22.7.1999).

### 2.2. Ultrafine Bubble Generation and Measurement

Air-filled UFB water was generated via a patent-pending (US 18/945,882) hydrodynamic cavitation process using equipment designed and fabricated by Hydrosome Labs, LLC (Westmont, IL, USA). The hydrodynamic cavitation process operates through controlled pressure differentials and flow dynamics to generate stable UFBs with reproducible size distributions. Quality control measures during UFB generation included real-time monitoring of flow rates, pressure parameters, and temperature, to ensure consistent production conditions across batches. UFB concentrations were measured using nanoparticle tracking analysis (ZetaView^®^ PMX-130-Z-MONO-488-F, Particle Metrix, Inning am Ammersee, Germany) immediately following water preparation for each batch. The ZetaView system utilizes laser light scattering and Brownian motion analysis to determine particle concentration and size distribution with a detection range of 10–2000 nm and concentration accuracy of ±10%. Measurements were performed in triplicate for each water batch, with the coefficient of variation maintained below 15% to ensure measurement reliability.

UFB water (bubbles filled with atmospheric air) and deionized water (control treatment) were supplied by Hydrosome Labs, LLC and stored at ambient temperature (21–29 °C). All water samples (UFB and control) were supplemented with minerals for taste and hydration purposes (MgCl_2_·6H_2_O, 15 mg/L; CaCl_2_·2H_2_O, 10 mg/L; NaHCO_3_, 10 mg/L). Following a 14-day acclimatization period, the rats were randomized into two groups of twelve animals each: the control group received deionized water, while the test group received UFB water. Water bottles were refilled every two days or as needed. UFB concentrations in the test group were intentionally increased at week 7 to investigate dose-dependent effects on biological parameters. This study design was chosen based on preliminary experiments suggesting that higher UFB concentrations might induce more pronounced biological responses.

**Figure 2:**
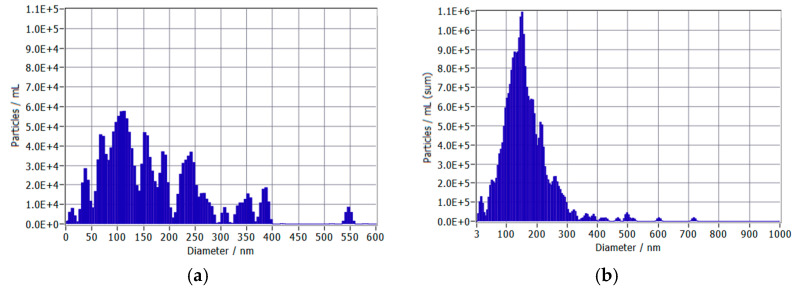
Distribution of ultrafine bubble diameters in two water samples: (**a**) Week 0–6 (Low UFB Phase) and (**b**) Week 7–12 (High UFB Phase).

Concentrations of UFBs in the test group were measured via nanoparticle tracking analysis (ZetaView^®^ PMX-130-Z-MONO-488-F, Particle Metrix, Germany). Concentrations and distributions of UFBs in the two lots are found in Table 1 and Figure 2. The size distribution histograms demonstrate that majority of the bubbles fall within the 100–300 nm range, typical of UFBs. The similar size distributions between lots (168 ± 100 nm vs. 164 ± 79 nm) confirm that the concentration increase at week 7 did not alter the UFB’s physical characteristics, allowing for the assessment of concentration-dependent effects independent of size variations. Holdback samples of each lot were tested at the conclusion of each phase of the study, and concentrations were not significantly different from freshly prepared UFB water samples.

#### 2.2.1. UFB Characterization Limitations

Several limitations in UFB characterization should be acknowledged. UFB concentrations were measured only at the time of water preparation for each batch and were not monitored throughout the study period. Temporal stability of UFB concentrations in water bottles during the 48 h replacement intervals was not assessed. Additional physicochemical properties including zeta potential, dissolved oxygen content, and UFB degradation kinetics were not measured. The reported UFB concentrations (1.7 × 10^6^ and 6.5 × 10^9^ UFBs/mL) represent the initial values at the time of water preparation, however holdback samples tested during the course of the study verified these levels were sustained.

### 2.3. Sample Collection and Analysis

Blood samples were collected via the retro-orbital sinus at weeks 0, 6, 8, and 12. For each animal, approximately 200 μL blood was collected in K2EDTA tubes for hematology and 200–300 μL in separate tubes for serum separation. Blood was centrifuged at 4000 rpm for 10 min at 4 °C to obtain plasma or serum. Hematology analysis was conducted using a GP BHA-3000VET automatic hematology analyzer (Nanjing, China) for parameters including hematocrit, hemoglobin concentrations, erythrocyte count, reticulocytes, total and differential leucocyte count, and platelet count.

Fresh fecal samples for microbiome analysis were collected at weeks 0, 6, 8, and 12 to align with blood sampling timepoints. Samples were collected in sterile containers and immediately frozen at −80 °C until analysis. Microbiome analysis was conducted using DNA extraction with QIAamp PowerFecal Pro DNA Kit (Venlo, The Netherlands), followed by V3–V4 16S rRNA gene sequencing using 341F (5′-CCTAYGGGRBGCASCAG-3′) and 806R (5′-GGACTACNNGGGTATCTAAT-3′) primers. Sequencing was performed on an Illumina NextSeq 2000 platform (San Diego, CA, USA) with 300 bp paired-end reads, and bioinformatic analysis utilized the QIIME2 pipeline (open-source software).

The SCFA analysis was performed via GC-MS using an Agilent 7890A (Santa Clara, CA, USA) system coupled with a 5975C mass selective detector. Compounds measured included acetate, propionate, butyrate, iso-butyrate, valerate, and iso-valerate. Serum was evaluated for lipid profiles using a Beckman Coulter AU480 (Brea, CA, USA), and inflammatory markers (IL-1β, TNF-α, and IL-10) were analyzed using commercial ELISA kits (SARD Biosciences, Mumbai, India).

### 2.4. Histopathological Analysis

Upon completion of the study, the animals were humanely euthanized using carbon dioxide asphyxiation. Complete gross necropsies were performed, including examination of external surfaces, all orifices, and cranial, thoracic, and abdominal cavities. The liver, kidneys, and colon were collected, weighed, and processed for histopathology. Tissues were fixed in 10% neutral buffered formalin, processed routinely for paraffin embedding, sectioned at 5 μm thickness, and stained with hematoxylin and eosin (H&E). Histopathological evaluation was performed by a board-certified veterinary pathologist blinded to the treatment groups.

### 2.5. Statistical Analysis

Statistical analyses were performed using SPSS Release (23.0 version). Results were expressed as mean ± S.E.M/SD. Differences between control and treatment groups were analyzed using independent sample t-tests. Statistical significance was set at *p* ≤ 0.05, with significance levels indicated as * *p* ≤ 0.05, ** *p* ≤ 0.01, and *** *p* ≤ 0.001. Given the high inter-individual variability commonly observed in gut microbiome studies, we also examined trends in the data to identify biologically relevant patterns that might not reach statistical significance due to limited power. Due to the exploratory nature of this study and the related biological pathways being investigated, multiple comparison adjustments were not applied. Future confirmatory studies should incorporate appropriate corrections for multiple testing. As detailed in Section 2.2.1, UFB characterization was conducted at water preparation, with holdback sample testing confirming the concentration stability throughout each study phase.

## 3. Results

### 3.1. Microbiome Analysis and Taxonomic Changes

**Figure 3:**
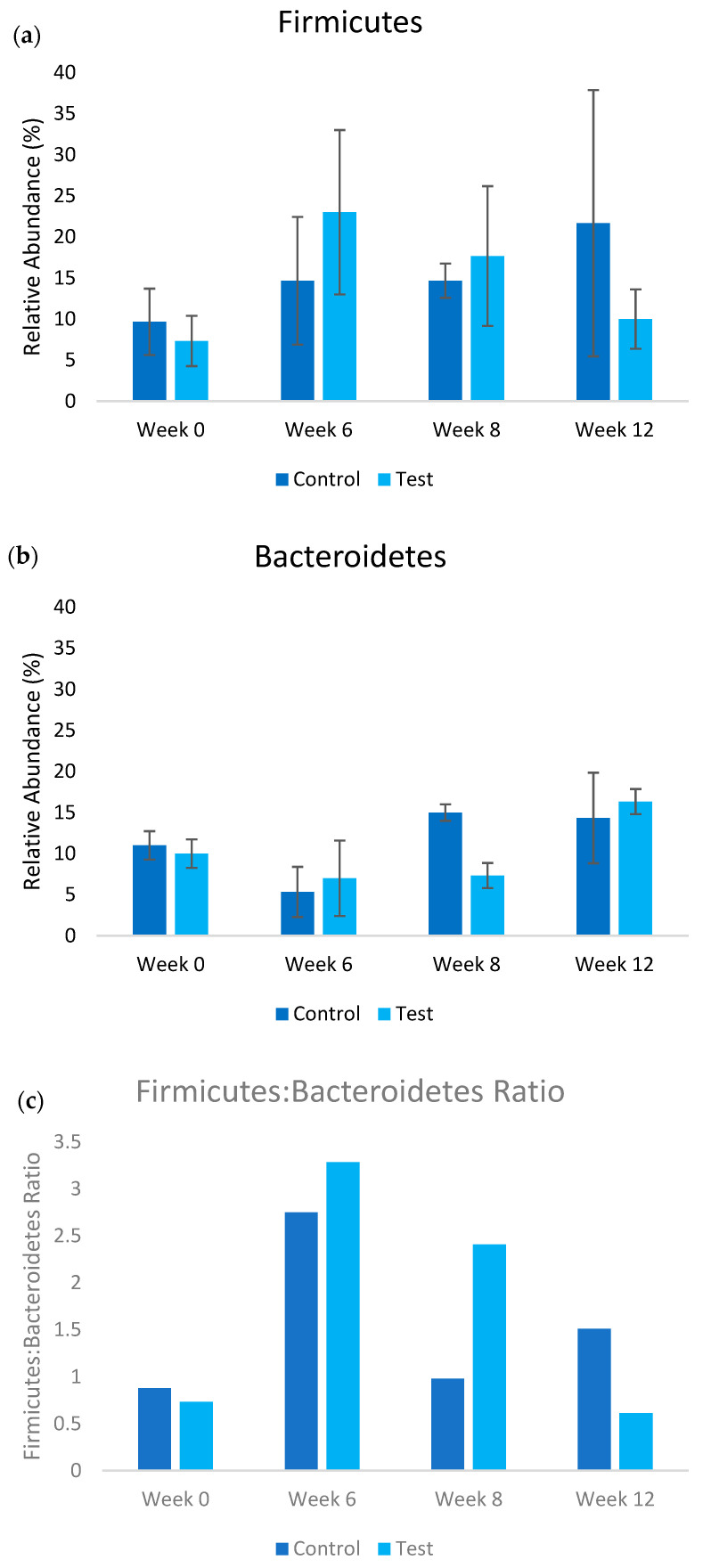
Relative abundance of (**a**) Firmicutes and (**b**) Bacteroidetes populations in test (UFB water treatment) and control treatments over the course of the study. The ratio of the Firmicutes–Bacteroidetes is shown in (**c**). In the test treatment, the concentration of ultrafine bubble water was increased post week 6.

The metagenomic analysis revealed distinct temporal shifts in gut microbiota composition between the control and test groups throughout the 12-week study period. Analysis focused on Firmicutes and Bacteroidetes as these phyla represent the dominant gut bacterial populations and their ratio indicates metabolic health status. At baseline (Week 0), both groups exhibited comparable Firmicutes-to-Bacteroidetes ratios (Figure 3), with secondary populations of Actinobacteria, Pseudomonadota, and Verrucomicrobiota present (see Supplementary Data). A significant transition occurred by Week 6, characterized by Firmicutes becoming the predominant phylum across both groups, followed by Bacteroidetes, Bacillota, and Actinobacteria. Following Week 6, coinciding with increased UFB concentration in the test group, divergent patterns emerged in the relative abundances of major phyla between groups (Figure 3). The test group demonstrated progressive increases in Bacteroidetes abundance (+5% Week 6–8; +122% Week 8–12; *p* = 0.16 and *p* = 0.14, respectively) accompanied by concurrent decreases in Firmicutes (−23% Week 6–8; −43% Week 8–12; *p* = 0.18 and *p* = 0.12, respectively). In contrast, the control group maintained relatively stable Bacteroidetes levels (−4% Week 8–12) while exhibiting increased Firmicutes abundance (+48% Week 8–12). While these microbial shifts did not reach statistical significance (*p* > 0.05), the consistent directional patterns and substantial effect sizes align with the statistically significant changes observed in SCFA production and inflammatory markers, suggesting biologically coherent responses to UFB treatment. The substantial variation in microbiome composition between individual rats reduces the statistical power to detect significant differences between groups, a characteristic challenge in microbiome research where inter-individual variability can account for 20–50% of compositional variation (beta diversity) and 10–30% of the variation in microbial community structure (unifrac distance) [34].

Importantly, current metagenomic techniques identified approximately 25–50% (averaging 25%) of the total gut microbiome population, indicating substantial uncharacterized genomic content. This limitation suggests potential additional microbial contributions to the observed treatment effects that warrant further investigation through advanced sequencing methodologies.

### 3.2. Short Chain Fatty Acid Production

**Figure 4:**
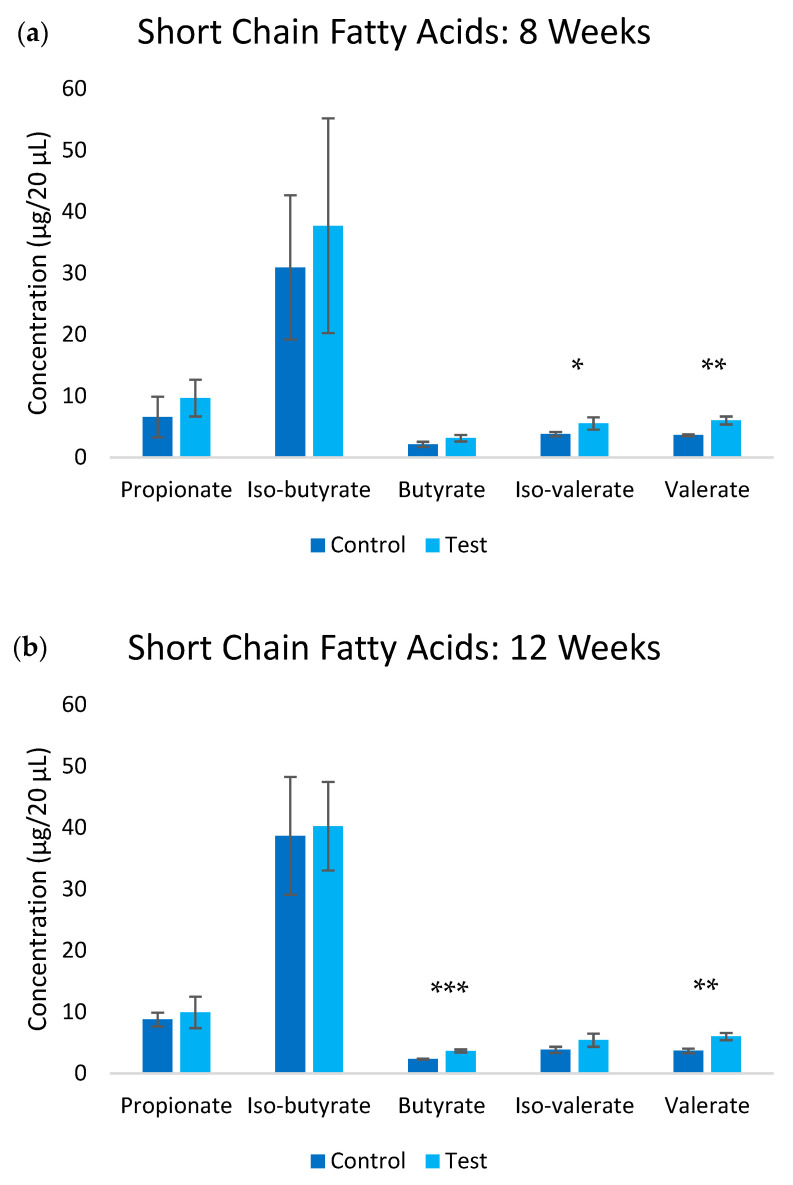
Short chain fatty acid levels in the blood at (**a**) 8-week and (**b**) 12-week timepoints. Significance * *p* ≤ 0.05, ** *p* ≤ 0.01, ***, *p* ≤ 0.001.

Treatment with UFB water (test group) induced substantial changes in the SCFA production profiles (Figure 4). Analysis in week 8 revealed significant increases across multiple SCFA metabolites in the test group compared to the control group. Iso-valerate levels increased by 45.3% (*p* ≤ 0.05), and valerate increased by 66.3% (*p* ≤ 0.01). These elevations were largely maintained throughout week 12, with butyrate (*p* ≤ 0.001) and valerate (*p* ≤ 0.01) showing the most sustained increases at 56.0% and 63.1%, respectively. Acetate levels remained below detection limits in both groups throughout the study period, suggesting specific modulations of SCFA metabolic pathways rather than a general increase in fermentation activity.

### 3.3. Inflammatory Markers

**Figure 5:**
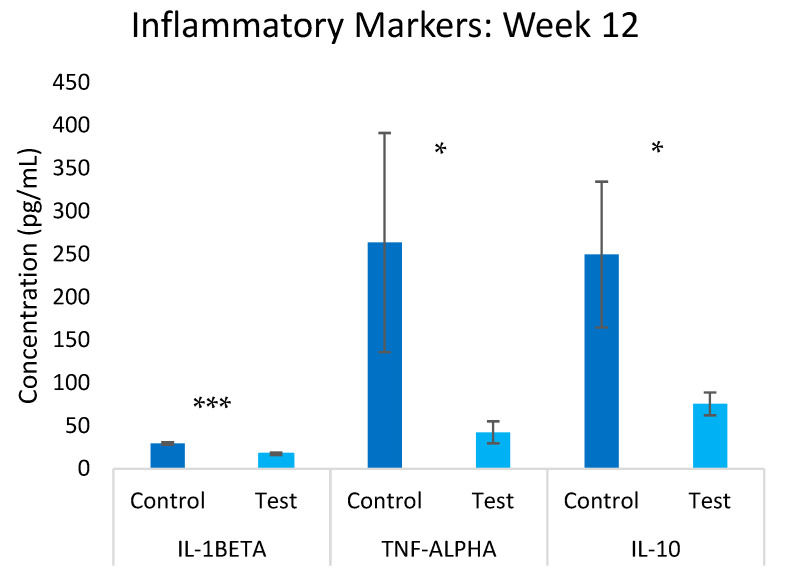
Overview of inflammatory markers in the test and control groups at the 12-week mark. Significance * *p* ≤ 0.05, *** *p* ≤ 0.001.

The inflammatory profile was significantly altered in response to the test group (Figure 5). By week 12, substantial reductions were observed in both pro-inflammatory and regulatory cytokines. IL-1β levels decreased by 41.0% (*p* ≤ 0.05) in the test group compared to the control group, while TNF-α showed the most dramatic reduction of 84.0% (*p* ≤ 0.05). Notably, the regulatory cytokine IL-10 also decreased by 69.8% (*p* ≤ 0.05). This comprehensive downregulation of inflammatory markers suggests a broad modulation of immune response pathways rather than a simple anti-inflammatory effect. The concurrent reduction in both pro-inflammatory cytokines and the regulatory cytokine IL-10 indicates a complex remodeling of the immune environment that requires further investigation. While this could reflect improved immune homeostasis, it may alternatively indicate immunosuppressive effects that could compromise immune surveillance functions. The clinical significance of this immune profile modulation remains unclear and warrants careful evaluation in future studies, particularly regarding the potential impacts on antimicrobial immunity and cancer surveillance as highlighted in the recent literature [35].

### 3.4. Physiological Parameters

Throughout the 12-week study period, no statistically significant differences were observed between the test and control groups in key physiological parameters including body weight gain, feed consumption, and water intake. Histopathological examination of the colon tissue revealed minimal focal degeneration and multifocal mononuclear cell infiltration in both groups, with these changes being considered, likely due to incidental or spontaneous background findings rather than treatment-related effects. The test group showed increased GALT activity at week 8 (see Supplementary Data), suggesting enhanced immune surveillance without pathological implications. These observations indicate that UFB supplementation was well-tolerated while inducing significant beneficial changes in gut microbiome composition and function.

This comprehensive analysis demonstrates that consumption of the UFB infused drinking water induces substantial changes across multiple parameters of gut health, including microbiome composition, metabolite production, and immune response, while maintaining physiological homeostasis. The concurrent nature of these changes suggests a coordinated biological response rather than isolated effects on individual parameters.

### 3.5. Concentration-Dependent Response Analysis

To evaluate the relationship between UFB concentration and biological outcomes, we compared response patterns between the low-concentration phase (weeks 0–6, 1.7 × 10^6^ UFBs/mL) and high-concentration phase (weeks 7–12, 6.5 × 10^9^ UFBs/mL). This analysis revealed systematic concentration-dependent responses characterized primarily by directional reversals across multiple biological systems (Table 2). Following the 3824-fold concentration increase at week 7, most parameters exhibited complete directional reversals rather than simple dose–response relationships. Butyrate levels shifted from −23.0% (low phase) to +56.2% (high phase), while TNF-α changed from +225.7% to −83.9%, indicating a transition from pro-inflammatory to anti-inflammatory effects. Similarly, valerate, IL-1β, IL-10, and Firmicutes all demonstrated directional reversals between the concentration phases. Bacteroidetes showed enhanced responses in the same direction (+31.3% to +133.3%, representing a 4.3-fold greater magnitude), while other parameters exhibited complete directional shifts. These systematic reversals occurred across metabolic (SCFAs), immune (cytokines), and microbial (phylum composition) systems, with effects typically detectable by week 8 (2 weeks post-concentration increase). The consistent pattern of directional reversals provides evidence for specific UFB biological activity rather than simple dose–response relationships, as such systematic changes across multiple biological systems cannot be attributed to experimental artifacts and indicate coordinated physiological adaptations highly sensitive to concentration thresholds.

The statistical robustness of these concentration-dependent effects is evidenced by the systematic nature of directional reversals across multiple biological systems. With 85.7% of parameters exhibiting directional reversals and 71.4% achieving statistical significance (*p* ≤ 0.05), these findings cannot be attributed to random variation or experimental artifacts. The coordinated timing of these effects, with most changes detectable by week 8 (2 weeks post-concentration increase), further supports the conclusion that UFB concentration increases trigger systematic biological threshold responses rather than gradual dose-dependent changes.

## 4. Discussion

This study demonstrates that UFB water modulates gut metabolism and reduces inflammatory markers, with effects showing distinct temporal patterns that differ markedly between the study phases. Importantly, the temporal variability observed in microbial composition changes requires cautious interpretation, as both Firmicutes and Bacteroidetes populations followed different trajectories during weeks 0–6 versus weeks 6–12. During the initial low-concentration phase (weeks 0–6), both groups showed convergent increases in Firmicutes abundance, suggesting adaptation to laboratory conditions or dietary factors independent of UFB treatment. However, following the concentration increase at week 7, divergent patterns emerged with the test group showing progressive Bacteroidetes increases (+122% weeks 8–12) and Firmicutes decreases (−43% weeks 8–12), while the controls maintained stable Bacteroidetes levels and continued Firmicutes increases (+48% weeks 8–12). While changes in the gut microbiota composition were observed between the test and control groups, particularly in Bacteroidetes and Firmicutes populations after high UFB concentration supplementation, the substantial proportion of uncharacterized microorganisms (50–75% of total gut microbiome population) suggests that the observed biological effects may involve currently unidentified microbial species and pathways [16,17]. This limitation in metagenomic identification, combined with typical inter-rat variations accounting for 20–50% of microbiome compositional differences, emphasizes the need for careful interpretation of microbial population shifts.

The most compelling findings emerged in the metabolic and inflammatory markers, particularly following the increase in UFB concentration after week 6. The substantial increase in initial UFB concentration (from 1.7 × 10^6^ to 6.5 × 10^9^ UFBs/mL at water preparation) preceded significant changes in SCFA production, notably butyrate and valerate (56.0% and 63.1% increases, respectively). The sustained elevation of these metabolites through week 12 indicates a stable modification of microbial metabolism rather than a transient effect, while the varying degrees of elevation among the different SCFAs suggests selective pathway modulations [19,21].

The systematic directional reversals observed across 85.7% of the measured parameters provide compelling evidence for UFB concentration-dependent biological thresholds rather than linear dose–response relationships. At low UFB concentrations (1.7 × 10^6^ UFBs/mL), limited surface area may primarily influence local microenvironmental conditions, potentially creating mild stress responses that trigger pro-inflammatory pathways. However, the 3824-fold concentration increase to 6.5 × 10^9^ UFBs/mL dramatically expands UFB surface area, enabling direct physical interactions with bacterial membranes and facilitating enhanced metabolite transport. This transition from indirect environmental modulation to direct bacterial interaction explains the systematic directional reversals, where pro-inflammatory effects (TNF-α +225.7%) transition to anti-inflammatory effects (TNF-α −83.9%) through fundamentally different mechanisms. The rapid onset (detectable within 2 weeks) supports direct metabolic pathway modulation consistent with UFB-mediated enhancement of existing bacterial fermentation capabilities, emphasizing the critical importance of precise UFB concentration control for therapeutic applications [18,21].

These results extend beyond previous studies by demonstrating coordinated changes across multiple biological parameters, particularly at higher UFB concentrations [27]. The concurrent reduction in both pro-inflammatory cytokines and the regulatory cytokine IL-10 indicates a complex remodeling of the immune environment that requires careful interpretation. While this pattern could reflect improved immune homeostasis, it may alternatively indicate immunosuppressive effects that could compromise essential immune functions. Further investigation through functional immune assays and long-term safety studies is necessary to determine the clinical significance of this immune profile modulation. Future research should focus on improving metagenomic identification techniques to better characterize the currently unidentified gut microbiota, understanding the dose-dependent nature of UFB effects, investigating the precise mechanisms of UFB–bacterial interactions in specific disease conditions where gut microbiome modulation could provide therapeutic benefits, and including both sexes to establish broader applicability given well-documented sex differences in gut microbiota composition and inflammatory responses.

## 5. Conclusions

This study demonstrates that UFB water significantly modulates gut microbiota metabolism and immune responses in a concentration-dependent manner. At higher initial concentrations (6.5 × 10^9^ UFBs/mL at water preparation), UFB water induced substantial increases in specific SCFAs (butyrate +56.0%, valerate +63.1%) while dramatically reducing inflammatory markers (TNF-α −84.0%, IL-1β −41.0%, IL-10 −69.8%), though the precise exposure levels throughout the study period remain uncertain due to limited UFB characterization. These effects showed a temporal association with shifts in microbiome composition, though different patterns between study phases require cautious interpretation. The high proportion of uncharacterized microbiota (50–75%) fundamentally limits mechanistic conclusions and suggests that primary effects may involve currently unidentified pathways. The temporal relationship between increased UFB concentration and enhanced biological effects, coupled with the sustained nature of these changes, indicates that the UFB water’s unique properties may create favorable microenvironmental conditions for beneficial host–microbe interactions. These findings establish foundational evidence that UFB water warrants further investigation for targeted gut microbiome modulation, while highlighting the need for advanced metagenomic techniques to fully elucidate the mechanisms underlying their biological effects.

Future research should focus on: (1) conducting systematic dose–response studies with multiple intermediate UFB concentrations between 10^6^ and 10^9^ UFBs/mL to identify precise biological threshold concentrations and optimal therapeutic ranges; (2) improving metagenomic identification techniques to characterize the currently unidentified gut microbiota; (3) investigating UFB effects in specific disease models where gut dysbiosis is implicated; (4) including both sexes to establish broader applicability; and (5) conducting controlled in vitro studies to establish direct UFB–microbe interactions. Clinical studies will be necessary to establish safety, efficacy, and optimal dosing in human populations before therapeutic applications can be considered. These directions would advance our understanding of UFB–microbiome interactions and potentially establish UFB water as a novel therapeutic approach for microbiome-related conditions pending clinical validation.

## Figures and Tables

**Table 1:** Comparison of ultrafine bubble sizes and concentrations between two lots of water used in the test group. Concentrations of ultrafine bubbles were increased at the midway point of the study (week 7) to observe possible dose–response effects.

Ultrafine Bubble Test Group Water Samples	Concentration (UFBs/mL)	Mean Size ± SD (nm)
Supplied Week 0–6	1.7 × 10^6^	168 ± 100
Supplied Week 7–12	6.5 × 10^9^	164 ± 79

**Table 2:** Comparison of biological responses between low and high UFB concentration phases (test vs. control).

Parameter	Low UFB Phase *	High UFB Phase **	Magnitude Change	*p*-Value ***
	Week 6: Test vs. Control	Week 12: Test vs. Control		
Butyrate change (%)	−23.0	+56.0	+79.0	<0.001
Valerate change (%)	−75.7	+54.2	+129.9	<0.01
TNF-α change (%)	+225.7	−83.9	−309.6	<0.05
IL-1β change (%)	+4.1	−41.0	−45.1	<0.05
IL-10 change (%)	+351.7	−69.8	−421.5	<0.05
Bacteriodetes change (%)	+31.3	+133.3	+102.0	>0.05
Firmicutes (%)	+56.8	−56.5	−113.3	>0.05

* Low UFB Phase: Week 6 test vs. control (1.7 × 10^6^ UFBs/mL). ** High UFB Phase: Week 12 test vs. control (6.5 × 10^9^ UFBs/mL). *** *p*-values from independent t-tests comparing test vs. control groups.

## Data Availability

Raw and Supplementary Data will be shared with MDPI during the submission process.

## Supplementary Materials

The following supporting information can be downloaded at: https://www.mdpi.com/article/10.3390/nano15151193/s1.

## Abbreviations

The following abbreviations are used in this manuscript:

UFB	Ultrafine bubble
HPLC	High performance liquid chromatography
GC	Gas chromatography
TNF	Tumor necrosis factor
IL	Interleukin
SCFA	Short chain fatty acids
GALT	Gut-associated lymphoid tissue
SD	Standard deviation
ELISA	Enzyme-linked immunosorbent assay
MS	Mass spectrometer
DNA	Deoxyribonucleic acid
