# Supplementary material for: Effects of Ultrafine Bubble Water on Gut Microbiota Composition and Health Markers in Rats
Source: Nanomaterials (Basel). 2025 Aug 5;15(15):1193. doi: 10.3390/nano15151193 (PMC12348793; doi:10.3390/nano15151193)
Supplement: Supplementary file 1 [file nanomaterials-15-01193-s001.zip › nanomaterials-3721151_Supplementary Data/SCFAs and Inflammatory Data Tables.pdf]

Table 6: ELISA-Cytokines Biomarker

Week	Parameter (pg/ml)	Animal Group	N	Mean	Std. Deviation	Std. Error Mean	P-value
Week 0	IL-1BETA	Vehicle control	3	29.149	2.983	1.722	ns
		Treatment Group	3	24.144	3.878	2.239	
	TNF-ALPHA	Vehicle control	3	426.403	104.473	60.318	**
		Treatment Group	3	76.057	38.506	22.231	
	IL-10	Vehicle control	3	312.136	107.812	62.245	*
		Treatment Group	3	94.866	32.239	18.613	
Week 6	IL-1BETA	Vehicle control	3	25.729	6.507	3.757	ns
		Treatment Group	3	26.796	5.130	2.962	
	TNF-ALPHA	Vehicle control	3	96.362	19.040	10.993	ns
		Treatment Group	3	313.852	249.861	144.257	
	IL-10	Vehicle control	3	66.059	47.025	27.150	ns
		Treatment Group	3	298.338	218.586	126.201	
Week 8	IL-1BETA	Vehicle control	3	23.510	2.394	1.382	ns
		Treatment Group	3	26.701	3.571	2.061	
	TNF-ALPHA	Vehicle control	3	218.353	300.801	173.667	ns
		Treatment Group	3	168.066	67.335	38.876	
	IL-10	Vehicle control	3	151.736	201.673	116.436	ns
		Treatment Group	3	151.270	66.253	38.251	
Week 12	IL-1BETA	Vehicle control	3	29.202	1.364	0.787	***
		Treatment Group	3	17.241	1.405	0.811	
	TNF-ALPHA	Vehicle control	3	263.406	127.686	73.719	*
		Treatment Group	3	42.268	12.861	7.425	
	IL-10	Vehicle control	3	249.439	84.979	49.063	*
		Treatment Group	3	75.428	13.254	7.652	

Key: *, **and *** Indicates statistical significant when compared with group G1, **ns:** Non significant

Table 7: Individual Animal Short Chain Fatty Acid Analysis by GC-MS

	Standard Details			Group 1						Group 2					
				A. No. 01		A. No. 02		A. No. 03		A. No. 13		A. No. 14		A. No. 15	
	Metabolite	Total Conc. (µg)	Std. Area	Area	Con. (µg/20 µl)	Area	Con. (µg/20 µl)	Area	Con. (µg/20 µl)	Area	Con. (µg/20 µl)	Area	Con. (µg/20 µl)	Area	Con. (µg/20 µl)
WK 0	Propionate	40	156916	57781	14.73	54321	13.85	56254	14.34	57279	14.60	64798	16.52	64004	16.32
	Isobutyrate	40	514481	0	0.00	19188	1.49	11201	0.87	14049	1.09	0	0.00	0	0.00
	Butyrate	40	5463964	0	0.00	56202	0.41	8435	0.06	0	0.00	19805	0.14	0	0.00
	Isovalarate	40	3603704	2215	0.02	0	0.00	0	0.00	0	0.00	11168	0.12	6603	0.07
	Valarate	40	5685677	4703	0.03	4400	0.03	2335	0.02	2887	0.02	17732	0.12	2961	0.02
	Standard Details			A. No. 04		A. No. 05		A. No. 06		A. No. 16		A. No. 17		A. No. 18	
	Standard Name	Total Concentration (µg)	Std. Area	Area	Con. (µg/20 µl)	Area	Con. (µg/20 µl)	Area	Con. (µg/20 µl)	Area	Con. (µg/20 µl)	Area	Con. (µg/20 µl)	Area	Con. (µg/20 µl)
WK 6	Propionate	40	156916	56161.0	14.32	65664.0	16.74	62139.0	15.84	43487.0	11.09	5979.0	1.52	55277.0	14.09
	Isobutyrate	40	514481	7710.0	0.60	0.0	0.00	0.0	0.00	0.0	0.00	0.0	0.00	0.0	0.00
	Butyrate	40	5463964	9436.0	0.07	17462.0	0.13	13907.0	0.10	17675.0	0.13	3884.0	0.03	9891.0	0.07
	Isovalarate	40	3603704	0.0	0.00	0.0	0.00	1131.0	0.01	0.0	0.00	1581.0	0.02	834.0	0.01
	Valarate	40	5685677	5507.0	0.04	9268.0	0.07	111810.0	0.79	3769.0	0.03	18761.0	0.13	8163.0	0.06

	Standard Details			A. No. 07		A. No. 08		A. No. 09		A. No. 19		A. No. 20		A. No. 21	
	Standard Name	Total Concentration (µg)	Std. Area	Area	Con. (µg/20 µl)	Area	Con. (µg/20 µl)	Area	Con. (µg/20 µl)	Area	Con. (µg/20 µl)	Area	Con. (µg/20 µl)	Area	Con. (µg/20 µl)
WK 8	Propionate	40	156916	36580.0	9.32	29550.0	7.53	11402.0	2.91	25867.0	6.59	38605.0	9.84	49195.0	12.54
	Isobutyrate	40	514481	546702.0	42.51	245121.0	19.06	401650.0	31.23	494554.0	38.45	255416.0	19.86	705046.0	54.82
	Butyrate	40	5463964	355105.0	2.60	244042.0	1.79	272953.0	2.00	362486.0	2.65	420274.0	3.08	506284.0	3.71
	Isovalarate	40	3603704	379767.0	4.22	329549.0	3.66	321154.0	3.56	422946.0	4.69	477411.0	5.30	597507.0	6.63
	Valarate	40	5685677	518195.0	3.65	491785.0	3.46	535581.0	3.77	752259.0	5.29	927441.0	6.52	890317.0	6.26
	Standard Details			A. No. 10		A. No. 11		A. No. 12		A. No. 22		A. No. 23		A. No. 24	
	Standard Name	Total Concentration (µg)	Std. Area	Area	Con. (µg/20 µl)	Area	Con. (µg/20 µl)	Area	Con. (µg/20 µl))	Area	Con. (µg/20 µl)	Area	Con. (µg/20 µl)	Area	Con. (µg/20 µl)
WK 12	Propionate	40	156916	29550.0	7.53	38065.0	9.70	35806.0	9.13	49695.0	12.67	29867.0	7.61	37494.0	9.56
	Isobutyrate	40	514481	355105.0	27.61	570246.0	44.34	567024.0	44.09	616301.0	47.92	504554.0	39.23	432395.0	33.62
	Butyrate	40	5463964	321154.0	2.35	330566.0	2.42	310555.0	2.27	517568.0	3.79	462486.0	3.39	521285.0	3.82
	Isovalarate	40	3603704	329549.0	3.66	311767.0	3.46	397677.0	4.41	598662.0	6.64	425679.0	4.72	441177.0	4.90
	Valarate	40	5685677	478519.0	3.37	511958.0	3.60	581951.0	4.09	889206.0	6.26	759522.0	5.34	916218.0	6.45

Note: A.No.- Animal Number; **STD. Area-** Standard Area**; ND** - Not detectable denotes that the concentration of the analyte is not within the detectable limits of the analysis performed. Standard curve has been plotted, and the concentrations of samples were analyzed against the standard curve. Acetate is not detected.
